# Characteristic Effects of the Cardiac Non-Neuronal Acetylcholine System Augmentation on Brain Functions

**DOI:** 10.3390/ijms22020545

**Published:** 2021-01-07

**Authors:** Yoshihiko Kakinuma

**Affiliations:** Department of Bioregulatory Science, Graduate School of Medicine, Nippon Medical School, Tokyo 113-8602, Japan; k12417853@nms.ac.jp; Tel.: +81-3-3822-2131 (ext. 5244)

**Keywords:** acetylcholine, heart, non-neuronal acetylcholine system, blood brain barrier, anti-inflammation, the vagus nerve

## Abstract

Since the discovery of non-neuronal acetylcholine in the heart, this specific system has drawn scientific interest from many research fields, including cardiology, immunology, and pharmacology. This system, acquired by cardiomyocytes independent of the parasympathetic nervous system of the autonomic nervous system, helps us to understand unsolved issues in cardiac physiology and to realize that the system may be more pivotal for cardiac homeostasis than expected. However, it has been shown that the effects of this system may not be restricted to the heart, but rather extended to cover extra-cardiac organs. To this end, this system intriguingly influences brain function, specifically potentiating blood brain barrier function. Although the results reported appear to be unusual, this novel characteristic can provide us with another research interest and therapeutic application mode for central nervous system diseases. In this review, we discuss our recent studies and raise the possibility of application of this system as an adjunctive therapeutic modality.

## 1. What Is the Non-Neuronal ACh System in the Heart?

Acetylcholine (ACh) is one of neurotransmitters in the autonomic nervous system (ANS), which is composed of the sympathetic and parasympathetic nervous systems (SNS and PNS, respectively). In the SNS, the primary neurons release ACh to activate the secondary neurons possessing nicotinic receptors for ACh and transduce the signals to them. The activated secondary neurons then release noradrenaline or norepinephrine from their terminal ends that binds to a specific receptor on the effectors to execute their specific physiological actions. In contrast, the PNS releases ACh from the terminal ends. For instance, the heart is innervated by the ANS, and the SNS exerts positive chronotropic and inotropic actions, including upregulation of its contraction, heart rate, and conduction velocity. In contrast, PNS decreases heart rate and conduction velocity. Therefore, ACh in the heart plays a role as a counterpart in these functions.

The distribution mode of nerve ends of the PNS in the heart, that is, the vagus nerve (VN), is completely distinct from that of the SNS. Compared to the SNS nerve ends, which are distributed to the entire cardiac ventricles, the PNS nerve ends are predominantly located at the sinus node and atrioventricular node, but are very sparsely distributed in the cardiac ventricles [1,2,3,4,5]. This finding distinctively provides us a cue to consider a novel system in the heart in terms of ACh synthesis independent of the PNS; that is, a non-neuronal ACh (NNA) synthesis in the heart or non-neuronal cardiac cholinergic system (NNCCS). In other words, ACh is synthesized by cardiomyocytes [6]. According to previous studies, it is broadly confirmed that cardiomyocytes can synthesize ACh with the machineries equipped with cardiomyocytes, including choline transporter (CHT1), choline acetyltransferase (ChAT), a crucial enzyme for ACh synthesis, and vesicular ACh transporter (VAChT), a storage vesicle including ACh responsible for exocytosis, whereas it is rapidly degraded by acetylcholinesterase [7,8,9,10,11]. Furthermore, even rat primary cultured cardiomyocytes in vitro, which were cultured for one week, synthesized ACh with sufficiently detectable levels by High Performance Liquid Chromatography (HPLC) [6]. Based on these pioneering studies independently conducted by other researchers, the concept of the NNA system in the heart or NNCCS has been established [6,7,8,9,10,11].

Afterwards, significant functional evidence regarding this system has been further accumulated to indicate that the system is not an accessory but indispensable for homeostasis of cardiac functions using ChAT gene knockout or knockdown method in vitro. For example, the system decreases cellular energy metabolism by reducing oxygen consumption not only in contracting cardiomyocytes but also in non-contracting cells [6,12]. Second, as implicated with the first issue, this system preferentially uses glucose as an energy substrate through the upregulation of a glucose transporter [13,14,15]. Third, it sustains cell–cell interaction and maintains protein expression of connexin 43 and β-catenin up to a necessary level [12]. Fourth, it sustains cellular resiliency against serum starvation, energy starvation, hypoxic insults, and norepinephrine exposure [9,10,11,12,13,14,16]. Lastly, it modulates immune responses in the injured heart by suppressing cytokine expression [17]. This cardiac finding was partly shared with further findings about other cells, including microglia [18], endothelial cells [19], macrophages [20] and immune cells [21,22,23], suggesting that NNA generally possesses anti-inflammatory effects. Taken together with all these experimental results, it should be concluded that the NNA in the heart or NNCCS represents a basic cardiac machinery to play a self-defensive role against overshooting stress.

## 2. A Model Mouse Representing the Activated Non-Neuronal ACh System in the Heart

### 2.1. Cardiac Phenotypes of the Transgenic Mouse

**Figure 1:**
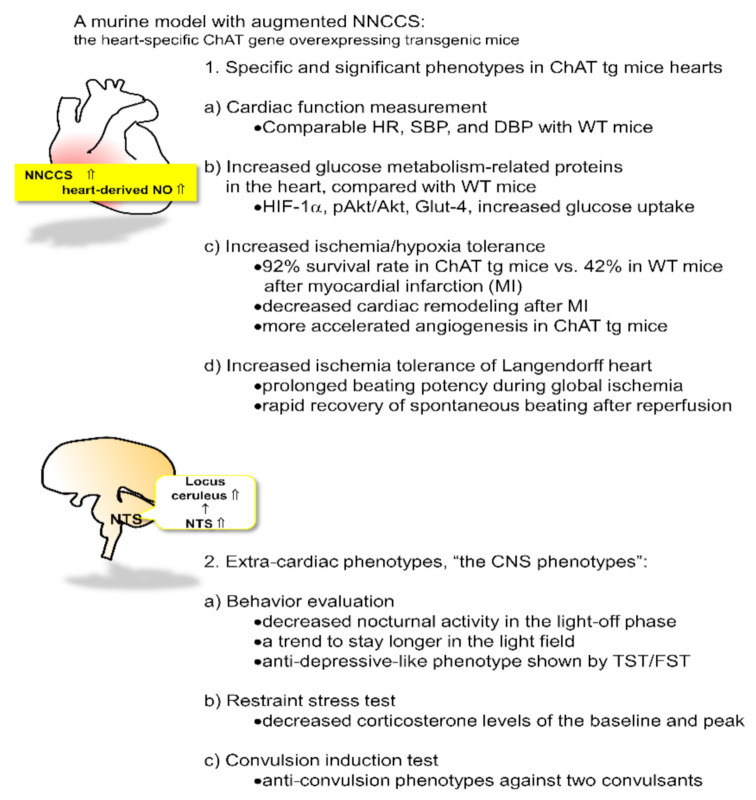
A murine model of the activated non-neuronal cardiac cholinergic system (NNCCS) with characteristic phenotypes of the cardiac and non-cardiac organ, the central nervous system (CNS).

To further investigate the physiological role of this system in the heart in vivo, we developed transgenic mice with the heart-specific overexpressing *ChAT* gene, a critical ACh synthesis enzyme gene, using the α-myosin heavy chain promoter, and labeled them as ChAT tg mice [14] (Figure 1). Another study modality was also conducted with deletions of the *VAChT*, a vesicle responsible for ACh storage and exocytosis, and *ChAT* genes, which clearly demonstrated completely opposite phenotypes to ours, leading us to the same conclusion regarding this system [16].

We consider that our ChAT tg mice represent a suitable model for the activation of this system because the transgene *ChAT* was confirmed to be expressed exclusively in the heart [14]. Although ChAT protein derived from the translated transgene was expected to be expressed both in atriums and ventricles [24,25], compared with the expression pattern of ChAT protein in wild-type (WT) mice, expression levels of the ChAT protein were increased predominantly in the ventricles. In contrast, atrial ChAT protein expression levels in ChAT tg were surprisingly not increased, as the ventricle ChAT expression did, to levels almost comparable with those in WT mice (unpublished data). ChAT tg mice showed comparable levels of blood pressure (both systolic and diastolic pressures, SBP and DBP, respectively) and heart rate (HR) with WT mice; therefore, systemic hemodynamic parameters in ChAT tg mice were not influenced by augmentation of this system [14,26].

Despite the comparable hemodynamic parameters between ChAT tg and WT mice, ChAT tg mice showed intriguing and striking cardiac phenotypes [14] that were also predicted by the in vitro phenotypes of the activated NNA system [12,13]. First, as the specific signal transduction following activated NNA synthesis in the heart, hearts of ChAT tg mice increased protein expression levels of HIF-1α, pAkt, and glut-4 as well as cardiac ventricular ACh levels, compared with those of WT mice, suggesting that even during normoxic conditions, ChAT tg mice upregulated the anti-hypoxic/anti-ischemic self-defense machinery in the heart. Therefore, ChAT tg mice with myocardial infarction (MI) survived more than WT mice in the chronic phase, with a survival rate of 92.3% (two weeks after the onset of infarction). In contrast, WT mice with MI usually died with cardiac rapture soon after MI, showing 41.7% survival rate [14].

Even after MI, the ChAT tg hearts possessed more intact myocardium but less fibrotic and necrotic changes. Furthermore, cardiac remodeling after MI was more suppressed in the heart of ChAT tg mice than in WT mice. Moreover, the hearts isolated from ChAT tg mice continued to beat for longer duration, even after perfusion-off in the Langendorff apparatus, than those from WT mice; alternatively, the former restarted to beat faster than the latter following reperfusion [14]. This observation clearly indicates that ChAT tg hearts are resilient to ischemia and efficiently sustain cardiac function even during insults.

### 2.2. Extra-Cardiac Phenotypes of ChAT tg Mice

As previously mentioned, ChAT tg mice were established to express the transgene restrictively in the heart. The heart-limited expression of ChAT was accurately verified by western blot analysis and immunohistochemical analysis of ChAT protein in each organ of ChAT tg mice. On the other hand, immunoreactive signals of ChAT in the ChAT tg mouse brain were completely comparable with those in WT mouse brains, and no neuronal cells with stronger signals of ChAT were detected in ChAT tg brains [26].

Despite the heart-limited transgenic mice overexpressing the *ChAT* gene, ChAT tg mice seemed more docile and quieter, and less aggressive than WT mice [26]. This characteristic phenotype was specifically observed when the mice were grasped. The presence of extra-cardiac phenotypes prompted us to further investigate whether ChAT tg mice possess other central phenotypes related to ChAT tg temperaments as follows [26].

(1) The total walking distance of ChAT tg mice was comparable with that of WT mice at daytime in a light-on phase; however, the nocturnal activity in the light-off phase was significantly decreased in ChAT tg mice compared with that in WT mice, specifically during the initial several hours following light-off. In general, this means that mice accelerate their activity in the light-off phase because they are nocturnal [27,28,29]. When they are transferred into a cage, they vigorously walk around to check whether the circumstance is safe or if they are under dangerous conditions. Therefore, decreasing nocturnal activity suggests that ChAT tg mice feel less anxious. Together with neither motor deficits nor muscular weakness in ChAT tg mice, these findings suggest that ChAT tg mice suffer from some influence on CNS function due to the augmented NNCCS [26].

(2) When subjected to a light and dark transition test, WT mice stayed longer in the dark field and they did not get out into a light field because they preferred dark conditions. However, ChAT tg mice tend to spend more time in the light field. These findings suggest that ChAT tg mice are less anxious. This may be compatible with decreased nocturnal activity [26].

(3) A representative test to evaluate a depressive-like phenotype includes the tail suspension test (TST) and forced swimming test (FST) [30]. In both tests, immobility time was measured during a part of the test because more prolonged immobility by giving up escaping or swimming indicates a more depressive-like phenotype in mice. When ChAT tg mice were subjected to both TST and FST, their immobility time was significantly decreased compared with that of WT mice. They continuously tried to escape from the suspension and to swim until the end of the tests. Furthermore, in elevated plus maze test, the total time spent by the ChAT tg mice in the open arms [31] was significantly longer than that of the WT mice; however, in the closed arms they stayed comparably with WT mice. Therefore, the decreased immobility time and increased time spent in the open space strongly suggest that ChAT tg mice were less under depressive-like conditions than WT mice [26].

(4) When WT mice were subjected to restraint stress with a silver metal net, they usually increased blood corticosterone concentration within 60 min because the stress activated the hypothalamus-pituitary-adrenal gland axis [32]. In contrast, when subjected to the same stress, ChAT tg mice increased up to only 50% of the peak blood corticosterone concentration in WT mice, suggesting that ChAT tg mice are resistant to stress and less stressed than WT mice [26].

(5) Finally, mice were stimulated with two convulsants, pilocarpine and pentylenetetrazole, which are well known to induce convulsions in animals [33,34]. We evaluated the susceptibility of convulsion by measuring duration, incidence, and survival rate from status epilepticus as well as by brain neuronal activity imaging using transcranial flavoprotein fluorescence imaging [35,36]. As predicted, when subjected to those convulsants, WT mice experienced convulsions with a higher incidence and longer duration, more often resulting in death due to status epilepticus. In contrast, ChAT tg mice less frequently experienced convulsions with a lower incidence and shorter duration. Significantly, more ChAT tg mice survived status epileptics than WT mice because neuronal activity of the ChAT tg mice brain evaluated by brain imaging was also significantly attenuated even in treatment with convulsants, compared with the WT mice brains [26].

## 3. The Mechanisms by Which Activation of the Non-Neuronal Cardiac Cholinergic System Influences the Brain Function

As mentioned above, ChAT tg mice are a representative useful model of activated NNCCS alone [14]; however, how does this heart-limited activated system influence the brain or CNS? The heart and CNS are connected via the VN, part of the PNS. As an effector organ, the VN is known to innervate the abdominal and intrathoracic organs in order to sense signals from other effector organs and execute orders from the CNS. The VN is composed of afferent and efferent fibers, the ratio of which is about 80% and 20% of the VN, respectively [37]. Consequently, the VN plays a role in the heart, mainly as an afferent fiber, in the transduction of various signals from the heart to the CNS. Based on these, we speculated that ChAT tg mice hearts transduce more signals from the heart to the CNS via the VN.

**Figure 2:**
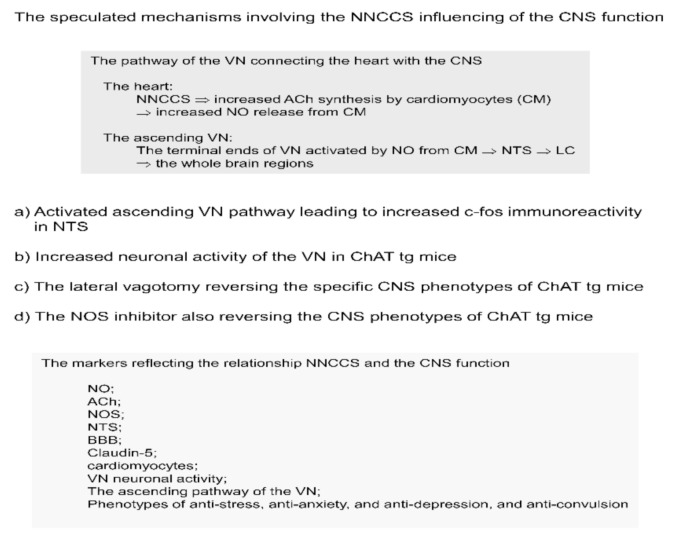
The speculated mechanisms involving the NNCCS which influences the CNS function.

(1) To assess this, the solitary tract nucleus of the brain stem was examined by immunohistochemistry using an anti-c-Fos antibody. The VN from the peripheral organs in the whole body terminates at the nucleus of the solitary tract (NTS), which is known as the center of the PNS and located at the dorsal part of the brain stem [38]. Different from directly measuring neuronal activity of the NTS, immunohistochemical analysis of c-Fos in the NTS is another tool to evaluate neuronal activation, with increased c-Fos immunoreactivity signals indicating activation of these cells [39]. With this c-Fos analysis, ChAT tg brain stem showed that the number of c-Fos positive neuronal cells around the NTS was significantly increased in ChAT tg mice compared with that in WT mice, indicating that the ChAT tg mice brain stem received more afferent signals of the VN, probably due to the activated NNCCS. In other words, the VN in ChAT tg mice may be activated to transduce afferent fibers triggered by the heart with activated NNCCS [26].

(2) To directly evaluate VN neuronal activity, the left ChAT tg mice VN was dissected and fitted with silver bipolar electrodes under anesthetic conditions. The frequency of neuronal firing signals was significantly higher in ChAT tg mice than in WT mice. This result indicated that ChAT tg mice VN hyperfunctioned with its elevated frequency, although it was not possible to conclusively state that the increased VN activity was derived mainly from afferent or efferent fibers [26].

(3) To examine whether the VN of ChAT tg mice was critical for representing the specific central phenotypes, ChAT tg mice were subjected to lateral (left) vagotomy because bilateral vagotomy usually caused death. Surprisingly, lateral vagotomy almost reversed the specific CNS phenotypes of ChAT tg mice; vagotomy reversed the anti-stress, anti-depressive-like, and anti-convulsion phenotypes of ChAT tg mice. These results clearly indicate that ChAT tg mouse CNS phenotypes are influenced by the afferent VN [26]. Taken together with the results thus far, the ChAT tg VN plays a crucial role in transducing the heart-derived signals to the CNS to induce significantly specific CNS phenotypes, that is, an anti-stress phenotype (Figure 2).

VN stimulation (VNS), a modality for influencing the VN function, has been known first as a method to treat drug-refractory uncontrolled epilepsy. This is accepted by the FDA as an adjunctive therapeutic tool for these patients [40,41]. Thereafter, VNS has been accepted to be used for patients with drug-refractory depression [42]. Moreover, it has been also applied to patients with chronic heart failure with sometimes beneficial effects, although the conclusion regarding VNS outcomes on chronic heart failure is contentious depending on clinical studies [43,44]. Then, we checked whether VNS-treated WT mice showed the same beneficial phenotypes as those in ChAT tg mice, that is, anti-stress, anti-depressive-like, and anti-convulsion phenotypes [45,46,47,48,49,50]. Finally, we confirmed that the VNS phenotypes of WT mice are all included in ChAT tg mice phenotypes. Therefore, these results prompted us to consider that ChAT tg mice may be subjected to so-called self VNS; however, the afferent fibers of ChAT tg mice should be predominantly activated in this specific case.

Which factor should be involved in triggering the activation of the VN afferent fibers in ChAT tg mice? ChAT tg mice hearts synthesized much more ACh by cardiomyocytes. Secreted ACh should bind to muscarinic receptors on cardiomyocytes, activating signal transduction, one of which is nitric oxide (NO) in response to ACh [51]. As also reported in our previous study, cardiomyocytes released NO soon after ACh treatment, which was blocked by atropine, a muscarinic receptor antagonist [52]. ChAT tg mice hearts produced significantly more NO than WT hearts [26]. Furthermore, NO has been reported to play a role as a neurotransmitter [53,54], however, via a non-receptor mediated fashion because NO is a gas-like substance.

To further investigate whether NO is critical for influencing VN activity, ChAT tg mice were treated with L-NAME, a NO synthase (NOS) inhibitor. As predicted, the NOS inhibitor significantly decreased VN activity frequency rapidly, indicating that intact NOS activity and production of adequate NO are indispensable for sustaining the VN activity. Our recent study supported the finding that NO derived from n (neuronal) NOS or NOS1 in the heart is critical for cardiac function and VN activity [55]. Based on these results, ChAT tg mouse heart-derived NO can trigger VN activation.

To further consolidate our speculation that NO derived from ACh synthesized by the activated system would be a trigger for VN activation, ChAT tg mice were treated with L-NAME and the CNS phenotypes were assessed. Intriguingly, the beneficial phenotypes were completely cancelled and reversed to those of WT mice. L-NAME completely suppressed such phenotypes as anti-stress, anti-depressive-like, and anti-convulsion phenotypes to levels comparable to those in WT mice [26]. These results clearly demonstrate that at least NO is one of the stimulators of VN nerve endings in the heart, causing activation of the VN afferent fibers. Furthermore, our recent study using heart-specific ChAT knockdown mice, which possessed a depressed NO content in the heart and downregulated VN activity, were susceptible to depressive-like phenotypes and more subjected to stress load [55].

## 4. Another Evidence of Potentiating the Non-Neuronal Cardiac Cholinergic System Involving Consolidation of Blood Brain Barrier

Thus far, we know that ChAT tg mice possess specific CNS phenotypes that are influenced by increased cardiac NO production and VN afferent activity. However, other than the anti-inflammatory effects of the cholinergic system, that are often reported and represented partly by the representative effects of VNS [56], it remains to be elucidated the mechanisms by which the CNS phenotypes of ChAT tg are influenced. We speculate that blood brain barrier (BBB) function may be more consolidated in ChAT tg because consolidation of the BBB, which is composed of tight junction components [57,58], can interfere with the extravasation of pro-inflammatory substances into the blood, resulting in anti-inflammatory responses in the brain. We further investigated CNS phenotypes of ChAT tg mice especially focusing on their BBB function [59].

(1) Initially, we performed Western blot analysis of the brain to evaluate the protein expression of claudin-5, one of the components of BBB [60,61,62,63]. Intriguingly, the ChAT tg brain expressed more claudin-5 protein than WT mice brains. This striking result prompted us to further examine the BBB function. As mentioned in our previous study, the beneficial CNS phenotypes are mediated by the VN afferent fibers of ChAT tg mice [13,26]; therefore, we checked the effects of lateral vagotomy on claudin-5 protein expression in the brain. Surprisingly, vagotomy caused downregulation of claudin-5 protein levels within 5 d following the vagotomy [59]. This also suggests the novel fact that the expression of claudin-5 in the brain is regulated by the VN, specifically the afferent fiber activation. In other words, here there is evidence of a tight link between the BBB and the VN [59].

The in vitro experiments also followed the in vivo features of BBB [62,64,65]. Murine brain endothelial cells from ChAT tg mice sustained enhancement of claudin-5 protein expression compared with those from WT mice. Furthermore, an in vitro reconstruction study of BBB using brain endothelial cells, astrocytes, and pericytes demonstrated that ChAT tg mice-derived reconstructed BBB was more resistant to Evans blue dye leakage than WT mice-derived reconstructed BBB [66]. These results clearly indicate that BBB function is more consolidated in ChAT tg mice than in WT mice via increased claudin-5 protein expression [59], although the precise mechanisms are still unknown. However, a recent study demonstrated that α7 nicotinic receptor stimulation increased claudin-5 expression in rat brain endothelial cells, and therefore, this may be considered one of the mechanisms [60,61].

(2) ChAT tg mice were subjected to brain cold injury, which is a BBB disrupting model [67,68,69]. The brain cold injury was developed using a cold metal cylinder with a diameter of 3–4 mm, which was attached to the surface of the right parietal bone strictly for 5 s. The effect of cold injury on BBB transient disruption peaked 24 h after injury, which was shown by the systemically distributed Evans blue dye. The area subjected to cold injury was restricted to blue color, indicating that the dye was extravasated into the brain parenchyma. With brain cold injury, WT mice brains showed remarkable blue staining in the surface of the injured brain; however, ChAT tg mice brains significantly attenuated Evans blue leakage, suggesting that the BBB function of ChAT tg mice was more strengthened and resistant to the injury [59].

Furthermore, the Parkinson’s disease model induced by 1-methyl-4-phenyl-1,2,3,6-tetrahydropyridine (MPTP), which is another model influencing BBB [70,71], was also applied to ChAT tg mice. Likewise, the brains of WT mice 3 d after injection of MPTP showed increased extravasation of sodium fluorescein into the brain parenchyma. In contrast, the ChAT tg mice brains significantly decreased the fluorescein levels in the brain. Although it was not fully studied regarding the mechanisms by which BBB consolidation leads to neuroprotective effects, ChAT tg brain was significantly resistant to neurodegeneration by MPTP, showing that more neuronal cells as well as neuron fibers were left intact in the substantia nigra, which is a main target of this model. These data clearly indicate that ChAT tg mice BBB was resistant to two different types of BBB disruption models [19].

Compatible with the more consolidated characteristics of ChAT tg BBB associated with increased immunoreactivity of claudin-5, the appearance of astrocytes in the ChAT tg brain was less hypertrophic and immunoreactive with glial fibrillary acidic protein (GFAP) staining [72], suggesting that less extravasating substances from the blood into the parenchyma attenuated reactive astrocyte transformation. This phenomenon was also evidenced by Western blot analysis of GFAP expression levels. The right brain of WT mice increased GFAP protein expression in response to the right injury compared with the left brain without injury; however, in the brains of ChAT tg mice, the laterality of the right to left brain GFAP expression was significantly decreased compared with that of the WT mice brains [59]. This is a novel and specific finding in ChAT tg mice brains caused by strengthened BBB function.

## 5. Anti-Inflammatory Reactions of ChAT tg Mice against Systemic Injection of LPS and Cold Brain Injury

As mentioned earlier regarding ChAT tg mice, their hearts produced more ACh. However, when LPS (10 mg/kg/dose i.p.) was systemically administered [73,74], ChAT tg mice survived more than WT mice within 48 h after injection, and their blood concentrations of TNF-α and IL-6 were significantly attenuated compared to those of WT mice. Moreover, Kupffer cells and the liver in ChAT tg mice also downregulated cytokines gene expression more than that in WT mice [59].

Likewise, in cold brain injury, the right injured parietal brain of ChAT tg mice expressed less cytokine gene expression than that of WT mice [59]. These results all support our concept that ChAT tg mice possess systemic anti-inflammatory response potency.

## 6. Speculated Underlying Mechanisms Responsible for Anti-Inflammatory Potency

Recent studies have reported that VNS, which was initially used as an anti-convulsion but later anti-heart failure or anti-depression treatment, plays a role in inhibiting inflammation. Since then, many research papers have come out to reveal that VNS attenuates inflammation, although the precise mechanisms remain to be fully investigated [43,75,76,77]. Initially, peripheral inflammation was the main target for VNS, including sepsis [56,78,79]; however, VNS later suppresses central brain inflammation [80,81], although the mechanisms may be complicated as the ascending root of the VN is not directly targeted to inflammatory lesions using the terminal ends. Rather, it is first terminated to the NTS followed by the second innervation to the whole brain, including the locus coeruleus (LC), thalamus, and hypothalamus [82]. The LC is an intermediate nucleus, which again projects third neurons into the whole brain as a noradrenergic neuron. In contrast, LC is also connected with the cholinergic pathway, which includes the prefrontal cortex [82].

ACh from the terminal ends of the VN exerts anti-inflammatory actions. Among receptors of ACh, including nicotinic and muscarinic receptors, the α7 nicotinic receptor has been well known to execute anti-inflammatory effects [83,84,85]. The α7 nicotinic receptor is unique with its homoheptamer structure, which is different from the other heterodimer nicotinic receptors [86]. The anti-inflammatory effects of the receptor may contribute to its signal transduction; however, compared with other nicotinic receptors, the α7 nicotinic receptor does not seem to utilize specific signal transduction modes that have already been reported [86]. However, the following characteristics of the receptor are speculated to be responsible for its anti-inflammatory function: the homomeric α7 nicotinic receptor desensitizes very rapidly, compared with other heteromeric receptors, with its agonists [79,86]. Furthermore, the α7 nicotinic receptor activates the JAK2/STAT3 pathway [87,88] as well as the Akt pathway [89,90], the former suppressing NF-κB translocation into the nucleus and the latter activating the Nrf2 pathway [90], which upregulates antioxidative factor HO-1 [91]. Based on these, its short opening time, rapid desensitization, activation of cellular protecting signals, or suppression of NF-κB nuclear translocation may be responsible for anti-inflammatory effects [86].

Likewise, the speculated mechanisms underlying upregulation of BBB claudin-5 remain to be studied in detail because there are few studies that have revealed a direct interaction between cholinergic nerve ends and brain endothelial cells composing the BBB. However, one in vitro study reported that the α7 nicotinic receptor agonist or glycogen synthase kinase inhibition upregulated tight junction proteins, including claudin-5 and occludin [61,92]. However, another study reported that the α7 nicotinic receptor was involved in attenuating the expression of BBB components [93,94]. The reason these results were contradictory remains to be elucidated. In contrast to the involvement of cholinergic agonists in regulating tight junctions, adrenergic receptors, instead of cholinergic receptors, were also reported to be responsible for sustaining BBB functions [27,95].

**Figure 3:**
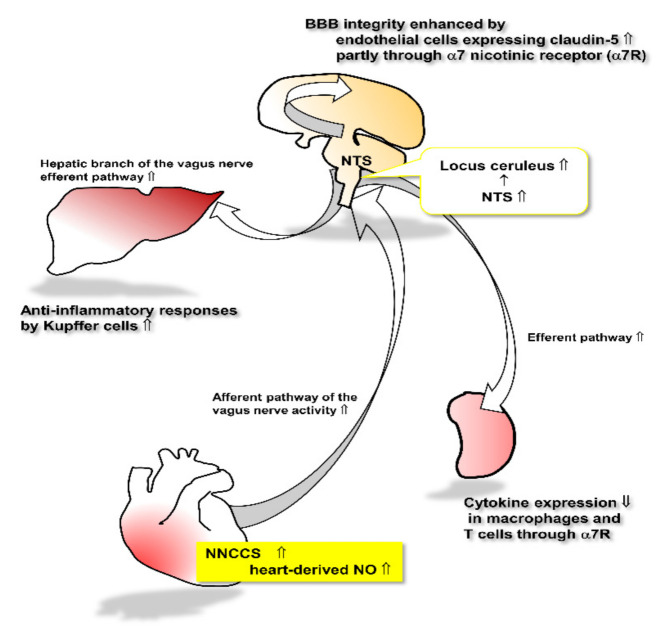
This schema represents the speculated link between augmented NNCCS and other organs.

However, our transgenic mice overexpressing the ChAT gene restrictively in the heart provide a novel significant clue; that is, the link between the activation of NNA in the heart and BBB consolidation as well as anti-inflammation, as comprehensively expressed in a schema (Figure 3). However, it remains to be determined whether these distinctive findings of upregulation of BBB function and anti-inflammatory response may contribute to only one or several factors. Therefore, further studies are needed to investigate and address these issues.

Despite the limitations of transgenic mice, the finding that the heart equipped with NNCCS influences higher levels of brain functions (e.g., mood and BBB) through the VN afferent pathway, may provide a novel concept to modulate the brain by the heart, not the brain–heart connection but heart–brain connection.

When the heart-derived non-neuronal ACh synthesis is activated, increased release of NO from cardiomyocytes stimulates the afferent pathway of the vagus nerve, leading to stimulation of the cholinergic and noradrenergic pathway in the brain through the NTS. The activated cholinergic system plays a role in increasing claudin-5 expression in brain endothelial cells, leading to the consolidation of BBB integrity. The efferent pathway of the vagus nerve, in turn, is activated to influence target organs including Kupffer cells in the liver as well as macrophages and T cells in the spleen. Those cells are regulated by the vagus nerve efferent pathway to attenuate inflammatory responses.

## 7. Concluding Remarks

Even though evidence of NNCCS or NNA in the heart has accumulated, there is no direct evidence of this system involving human diseases. Additionally, no answers have been found as to whether this system can be used as a therapeutic modality [10,96,97]. However, there are several studies to remind us of the possibility of addressing this system in human diseases. Donepezil, an Alzheimer’s disease drug known as an acetylcholinesterase inhibitor, can decrease the incidence and mortality of cardiovascular diseases in patients with Alzheimer’s disease [98,99,100]. Our study has already reported that donepezil upregulates NNCCS as an inducer of this system [4]. These results strengthen the speculation that the brain and heart are profoundly cross-talked through influencing each other, and the possibility of intervention in this system for both targets. Further studies focusing on this issue are anticipated.

## Data Availability

Data available on request due to restrictions e.g., privacy or ethical.

## Abbreviations

ACh	Acetylcholine
ANS	Autonomic nervous system
SNS	Sympathetic nervous system
PNS	parasympathetic nervous system
NNA	a non-neuronal ACh
NNCCS	non-neuronal cardiac cholinergic system
CHT1	choline transporter
ChAT	choline acetyltransferase
VAChT	vesicular ACh transporter
TST	tail suspension test
FST	Forced swimming test
NTS	The nucleus of the solitary tract
NO	Nitric oxide
NOS	NO synthase
VN	vagus nerve
WT	wild type
VNS	Vagus nerve stimulation
BBB	blood brain barrier
LC	locus coeruleus
